# Hemolysis risk after packed red blood cells transfusion with infusion pumps*

**DOI:** 10.1590/1518-8345.2625.3053

**Published:** 2018-10-11

**Authors:** Ana Maria Miranda Martins Wilson, Maria Angélica Sorgini Peterlini, Mavilde da Luz Gonçalves Pedreira

**Affiliations:** 1 Universidade de São Paulo, Escola de Enfermagem, São Paulo, SP, Brazil.; 2 Universidade Federal de São Paulo, Escola Paulista de Enfermagem, São Paulo, SP, Brazil.

**Keywords:** Infusion Pumps, Erythrocytes, Hemolysis, Evidence-based Nursing, Blood Transfusion, Patient Safety

## Abstract

**Objective:**

To evaluate the hemolysis biomarkers of packed red blood cells transfused by two different linear peristaltic infusion pumps at two infusion rates.

**Method:**

An experimental and randomized study was designed simulating the clinical practice of transfusion. Two linear peristaltic infusion pumps from different manufactures were studied in triplicate at 100 mL/h and 300mL/h infusion rates. The chosen hemolysis biomarkers were total hemoglobin, free hemoglobin, hematocrit, potassium and degree of hemolysis. They were analyzed before and after each infusion.

**Results:**

Potassium showed statistically significant variations in all scenarios of the experiment (P<0.010). In a separated analysis, potassium increased mainly at 300mL/h rate (P=0.021) and free hemoglobin had significant variation when comparing infusion pumps from different manufacturers (P=0.026). Although hematocrit, total hemoglobin and degree of hemolysis had increased after infusion, no statistically significance variations were identified.

**Conclusions:**

Hemolysis risk induced by a linear peristaltic infusion pump was identified by an increase in free hemoglobin and potassium markers. As the potassium biomarker is often increased in aged packed red blood cells, we do not recommend using them in this scenario. Additional studies should be performed about other markers and using larger samples in order to reinforce the transfusion practice in nursing.

## Introduction

Packed red blood cell (RBC) transfusion is often indicated as supportive care in different clinical scenarios of anemia as a purpose of increasing the oxygen-carrying capacity of erythrocytes^1^ ^-^ ^2^. Hemolysis is one of the clinical complications from blood transfusion therapy and it is related to functional reduction of hemoglobin. Red cell damage may release potassium and free hemoglobin to the patient plasma, possibly causing clinical complications in renal and cardiovascular systems^3^ ^-^ ^4^. 

Packed RBC is often delivered by gravitational infusion sets and in some scenarios with additional aid of positive or negative external pressures sets^5^ ^-^ ^6^. However, the safety of these pressure devices is not clear, once there are several factors that can influence erythrocyte integrity^3^ ^,^ ^6^.

Infusion pumps (IPs) are among the most commonly used devices for controlling fluid infusion. Several models of IPs are available in the market with different propulsion mechanisms, such as peristaltic, shuttle, piston cassette, syringe and diaphragm^6^ ^-^ ^7^.

Electronic infusion devices have brought innovation to the intravenous therapy through rigorous fluid controlling, security in pressure and air alarms; however, the concern with the safety of this equipment in blood transfusions remains^3^ ^-^ ^4^ ^,^ ^8^. The IPs mechanism can also be influenced by the infusion rate, age of RBC and preservative solution, different types of intravenous sets, needle gauge and in-line filters^7^ ^-^ ^8^.

The existing evidence does not precisely define the result of the mechanical actions of infusion devices over the red blood cells. Also, there are few and conflicting studies about the impact of using IPs on the integrity of RBCs^8^.

Peristaltic mechanisms were evaluated in some studies and the results revealed major risk of hemolysis^8^ ^-^ ^12^. However, other studies with linear peristaltic IP showed low risk of hemolysis and concluded that the evaluated IPs were suitable for transfusion when considering limit of the degree of hemolysis at 0.8%, recommended by Brazilian, European and Canadian regulatory agencies^13^ ^-^ ^16^.

Infusion rate is also a variable mentioned in different studies, and it may have effect on the integrity of RBCs. Researchers have observed higher degree of hemolysis when there are higher RBC infusion rates in peristaltic IPs^17^ ^-^ ^18^. 

Volumetric linear IPs have been widely used in health services in Brazil and are provided by many manufacturers. Decision-making in the nursing area supporting the use of IPs in RBC transfusion is based on the information provided by the manufactures, but such facts are not consistently evidence-based and are rarely presented on the device manuals.

The aim of the current study was to evaluate the hemolysis biomarkers of packed RBCs transfused by two different linear peristaltic infusion pumps at two infusion rates.

## Method

This experimental study was performed at the Nursing Experiments Laboratory (LEEnf) after approval from the Research Ethics Committee of the Federal University of São Paulo (no. 56518/12).

The hemolysis risk was prospectively evaluated in three moments: control samples 1 (C1), taken from the blood bags before the experiment had started; control samples 2 (C2), collected after the infusion lines were filled; and the last phase, with the infusion velocity (V), collected after infusion on IPs in the selected rate. For post-infusion analyses, the blood volume within the infusion lines was discarded so that there was no interference in the free flow filling on post-infusion samples. The chosen biomarkers for hemolysis outcome were hematocrit (%), total hemoglobin (g/dL), free hemoglobin (g/dL), degree of hemolysis (%) and potassium (mmol/L).

Four packed RBCs units were necessary for the experiment. The amount of packed RBCs was calculated based on the number of analyses to assess the five markers during the study phases, resulting in 180 analyses.

The included devices were from two different manufacturers, namely infusion pump A (IPA), with horizontal peristaltic system, and B (IPB), with vertical peristaltic system. Each IP manufacturer was analyzed in triplicate, with a total of six devices evaluated, and the IPs were randomized according to the rate. The chosen rates simulated transfusions in adults within 4 hours and rapid transfusion, as in emergency situations, that is, 100mL/h and 300mL/h, respectively. Infusion accessories, such as blood-filtering kits and infusion extenders, were suitable for the equipment, as indicated by the manufacturers of the IPs.

In experiments with IPA, blood-filtering kits were suitable to the IP, with a total length of 230 centimeters (cm), and 16mL was the volume needed to fill the lumen.

For the IPB, there were no corresponding blood-filtering kits supplied by manufacturer. Therefore, the manufacturer recommended the use of extension tubes from other manufacturers, which were connected to the device for blood components. The total length of the infusion lines, including the extender and the indicated device, totaled 270cm, being 26mL the volume needed to fill the lumen.

The devices were randomized at each infusion rate and each packed RBC was directed to three IPs.

The four packed RBC were A+ type and were preserved within citrate, phosphate, dextrose and adenine (CPDA-1) anticoagulant solution. The storage time varied from 19 to 30 days. Regarding the exposition time of the RBC to the environment in the experimental phase, the higher amount was 176 minutes, found at 100 mL/h flow in IPB.

Before the experiments, the packed RBCs were stored at 2º C to 6º C and during the experiments it varied from 21.1ºC to 25.3 ºC in the experiments with IPA, and from 19.3º C to 24.1º C in those with IPB, monitored through infrared thermometer.

*In vitro* measurements were analyzed according to the suitable biomarker evaluation technique. Hematocrit assessment was conducted through centrifugation and, subsequently, the percentage result was set and double-checked through reading in a specific ruler. The spectrophotometry and the colorimetry assessment techniques were applied to the markers total hemoglobin, free hemoglobin, degree of hemolysis and potassium using the SP-22 Digital Spectrophotometer, Bioespectro^®^ brand, in a range of 325 nanometers (nm) to 1000 nm.

Other variables related to blood components and environmental conditions in the laboratory were measured and controlled, such as the storage time, temperature and exposure time to environment, laboratory temperature and air humidity. 

Statistical analyses were performed using the Statistical Package for the Social Sciences (SPSS) software, version 20.0. Variables were pre-tested for equal standard deviations and were estimated to follow a Gaussian distribution according to the Kolmogorov-Smirnov test. The distribution of these outcomes was examined using histograms. Normally distributed data were presented as mean ± standard deviation (SD), whereas non-normally distributed data were presented as medians and interquartile range (IQR).

Hemolysis markers were assessed by repeated measures analysis of variance test (ANOVA) and the Bonferroni post hoc test to discover which specific means differed one from another. A *P* value less than 0.05 (*P*< 0.05) was considered statistically significant.

## Results

This study covered the assessment of five markers in the different phases of the experiment conducted in each device (IPA and IPB); and in the rates of 100 mL/h and 300 mL/h selected in each IP.

The outcome variables are regarded in Table 1 that shows the variation of hemolysis biomarkers.

**Table 1:** Hemolysis biomarkers of packed red blood cells (RBC) administered by infusion pump A (IPA) and infusion pump B (IPB), according to the infusion rates of 100 milliliters per hour (mL/h) and 300 mL/h. São Paulo, SP, Brazil, 2015

	Experiment					Total
Variable		IPA* 100 mL/h^‡^	IPA* 300 mL/h^‡^	IPB^†^ 100 mL/h^‡^	IPB^†^ 300 mL/h^‡^	
Ht^§^ (%)	Mean ± SD^||^	71.3±2.2	68.3±1.3	72.9±2.5	68.4±0.9	70.2±2.6
	Min-Max^¶^	70-75	67-70	70-76	67-70	67-76
Total Hb** (g/dl^††^)	Mean ± SD^||^	24.4±1.7	26.1±2.4	25.1±1.8	27.1±2.0	25.7±2.2
	Min-Max^¶^	22.9-28.6	22.8-30.8	23.5-28	23.9-29,8	22.8-30.8
Free Hb** (g/dl^††^)	Mean ± SD^||^	0.160±0.152	0.444±0.170	0.258±0.147	0.368±0.203	0.308±0.201
	Min-Max^¶^	0.046-0.458	0.206-0.651	0.047-0.395	0.206-0.694	0.046-0.694
Degree of hemolysis (%)	Mean ± SD^||^	0.16±0.14	0.55±0.24	0.26±0.14	0.43±0.24	0.35±0.24
	Min-Max^¶^	0.06-0.40	0.20-0.81	0.06-0.38	0.23-0.83	0.06-0.83
Potassium (mmol/L^‡‡^)	Mean ± SD^||^	39.2±2.2	39.1±2,5	41.5	40.9±2.4	40.2±2.5
	Min-Max^¶^	35.6-43.7	35.8-42.9	37.6-44.5	37.4-42.9	35.6-44.5

*IPA- Infusion Pump A; †IPB-Infusion Pump B; ‡ ml/h-milliliters per hour; §Ht- Hematocrit; ||SD- Standard Deviation; ¶ Min-Max- Minimum- Maximum; **Hb- Hemoglobin; ††g/dL- grams per deciliter; ‡‡mmol/L-milimol per liter

Table 1 shows that hematocrit varied from 67% to 76%, and that the lowest hematocrit concentration was found in the 300mL/h infusion rate, in both devices. Total hemoglobin presented mean variation from 22.8g/dL to 30.8g/dL in experiments conducted with IPA, whereas the variation went from 23.5g/dL to 29.8g/dL in the experiment conducted with IPB.

Free hemoglobin presented values from 0.046g/dL to 0.651g/dL in the IPA, and from 0.047g/dL to 0.694g/dL in the IPB, thus reaching higher levels in the rate of 300ml/h, both in the IPA and IPB. 

The minimum degree of hemolysis found in the experiment phases with IPA was 0.06%, at infusion rate of 100mL/h; the maximum degree of hemolysis was 0.81%, found at infusion rate of 300mL/h. The degree of hemolysis in the IPB have varied from 0.06%, in the experiment phases at infusion rate of 100 mL/h, and 0.83%, at infusion rate of 300mL/h, which corresponded to the moment C1 at the data collection time. Potassium presented minimum value of 39.1mmol/L and maximum value of 43.7mmol/L in the IPA; and minimum of 37.4mmol/L and maximum of 44.5mmol/L in the IPB.

The hematocrit percentage showed non-parametric distribution behavior in the distribution normality analysis of the variables according to Kolmogorov-Smirnov test*.*

The effect of infusion system (C2) and infusion pump (V) on the RBC (C1), regardless of the flow and IP, are summarized in Table 2.

**Table 2:** Variation of packed red blood cells (RBC) hemolysis markers according to central and dispersion trend measures during the experiment phases. São Paulo, SP, Brazil, 2015

	Experiment phases				
Marker	C1*	C2^†^	V^‡^	Mean difference C1* and V^‡^ (CI^§^ 95%)	P
Ht^*||*^ (%)	70.08 (67-75)	70.25 (67-76)	70.40 (67-75)	0.32 (-1.24 to 0.57)	0.705^¶^
Total Hb** (g/dL^††^)	24.60 ±1.71	26.32 ±2.35	26.09 ±2.19	-1.48 (-3.07 to 0.10)	0.267^‡‡^
Free Hb** (g/dL^††^)	0.298 ±0.20	0.312 ±0.21	0.313 ±0.20	-0.01 (-0.04 to 0.01)	0.064^‡‡^
Degree of hemolysis (%)	0.35 ±0.25	0.35 ±0.24	0.35 ±0.24	0.006 (-0.03 to 0.05)	0.744^‡‡^
Potassium (mmol/L^§§^)	39.5 ±2.0	40.1 ±3.03	40.9 ±2.5	-1.39 (-2.28 to -0.48)	<0.010^**‡‡**^

* C1- Control samples 1- collected from the packed red blood cell bag; †C2- Control samples 2-collected after the infusion lines were filled; ‡V- Velocity Sample- collected after infusion on IP in the selected rate; §CI- Confidence Interval; || Ht -Hematocrit; ¶|Friedman test (mininum -maximum); ** Hb- Hemoglobin; ††g/dL- grams per deciliter; ‡‡Repeated measures ANOVA (Linear contrast between matters); §§ mmol/L- milimol per liter.

The percentage of hematocrit, the total hemoglobin concentration, the free hemoglobin and the degree of hemolysis did not present significant variation in the three moments of the experiment, regardless of IP and infusion rate. Despite of the statistical significance, free hemoglobin increased from 0.298±0.20g/dL in C1, 0.312±0.21g/dL in C2, and 0.31±0.20g/dL in V (*P*=0.064). Potassium concentration increased during the experiment from 39.5±2.0 mmol/L in C1; 40.1±3.03 mmol/L, in C2; 40.9±2.5mmol/L, in V (*P* < 0.01). 

**Figure 1:**
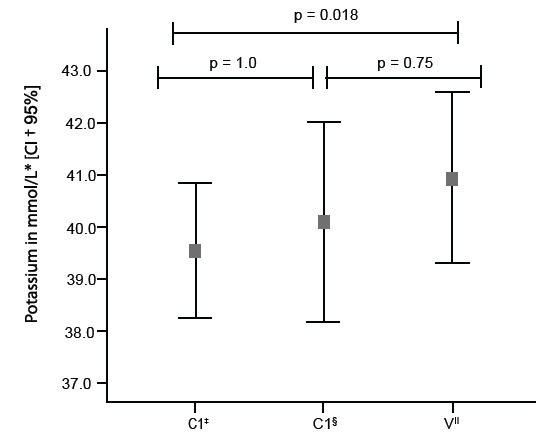
Potassium measured in the packed red blood cells in multiple paired comparisons (Bonferroni post hoc test) in the experiment phases: control sample, collected in the packed red blood cells bag (C1); the infusion system (C2); and the after infusion (V).

Multiple comparisons were performed with potassium in the three moments of the experiment according to the Bonferroni method, as showed in Figure 1.

* mmol/L- milimol per liter; †CI- Confidence Interval; ‡C1-Control samples 1- collected from the packed red blood cell bag; §,C2- Control samples 2-collected after the infusion lines were filled; ||V- Velocity Sample- collected after infusion on IPs in the selected rate.

These multiple comparisons applied to potassium concentration showed statistically significant increase between the control samples of the packed RBC and post-infusion samples in all infusion rates (*P* =0.018). These results, shown in Table 2 and Figure 1, indicate the potassium was significantly increased by the IPs.

The level of hemolysis markers of RBC was assessed according to the IPs (IPA and IPB) and infusion rates (100 and 300 mL/h), as shown in Table 3.

**Table 3:** Hemolysis markers of packed red blood cells (RBC) according to the central and dispersion trend measures applied to the control sample, collected in the packed red blood cells bag (C1); the infusion system (C2); and the after infusion (V), according to the flows 100 mL/h and 300 mL/h. São Paulo, SP, Brazil, 2015

	Experiment phases					
Markers	Flow mL/h*	C1^†^	C2^‡^	V^§^	Mean differences C1^†^ and V^§^	P-value
Ht^¶^ (%)	100	70.08 (70-75)	70.25 (70-76)	70.40 (70-75)	(CI^||^ 95%)	0.905**
	300	70.08 (67-70)	70.25 (67-70)	70.40 (67-70)	-0.50 (-1.94 to 0.94)	0.497**
Total Hb^††^ (g/dL^‡‡^)	100	23.83 ±0.77	25.48 ±2.23	24.89 ±1.83	-1.05 (-2.80 to 0.68)	0.180^§§^
	300	25.37 ±2.10	27.16 ±2.35	27.29 ±1.93	-1.92 (-5.32 to 1.48)	0.207^§§^
Free Hb^††^ (g/dL^‡‡^)	100	0.192 ±0.15	0.220 ±0.19	0.215 ±0.17	- 0.03 (-0.04 to 0.02)	0.068^§§^
	300	0.403 ±0.21	0.404 ±021	0.410 ±0.19	0.02 (- 0.07 to 0.05)	0.78^§§^
Degree of hemolysis (%)	100	0.21 ±0.15	0.22 ±0.17	0.22 ±0.15	-0.08 (-0.22 to 0.06)	0.209^§§^
	300	0.50 ±0.26	0.48 ±0.25	0.48 ±0.25	0.02 (-0.08 to 0.12)	0.617^§§^
Potassium (mmol/L^||||^)	100	39.6 ±1.4	40.2 ±3.6	41.3 ±2.8	- 1.76 (-3.73 to 0.20)	0.069^§§^
	300	39.5 ±2.7	40.0 ±2.7	40.5 ±2.5	-1.87 (-1.78 to -0.22)	0.021^**§§**^

*mL/h- milliliters per hour; † C1-Control sample 1- collected from the packed red blood cell bag; ‡C2- Control samples 2-collected after the infusion lines were filled; § V-Velocity Sample- collected after infusion on IP in the selected rate; ||CI- Confidence Interval; ¶Ht- Hematocrit; **Friedman test (minimum -maximum) ; ††Hb- Hemoglobin; ‡‡g/dL- grams per deciliter ; §§Repeated measures ANOVA (Linear contrast between matters; |||| mmol/L- millimol per liter.

Potassium concentration increased regardless on the programed infusion rate, but with a significant increase on experiments at rate of 300mL/h (*P*=0.021). The other markers did not present statistically significant variation regardless of the infusion rate.

The five selected markers were assessed in the experiment phases according to the IP, as shown in Table 4.

**Table 4:** Hemolysis markers of packed red blood cells (RBC), according to the central and dispersion trend measures applied to the control sample, collected in the packed red blood cells bag (C1); the infusion system (C2); and the after infusion (V), according to the infusion pump A (IPA) and the infusion pump B (IPB). São Paulo, SP, Brazil, 2015

	Experiment phases					
Markers	IP*	C1^†^	C2^‡^	V^§^	Mean differences C1^†^ and V^§^ (CI^||^ 95%)	p-value
Ht^¶^ (%)	IPA**	70.08 (67-72)	70.25 (67-75)	70.40 (67-75)	-0.50 (-1.78 to 0.78)	0.368^††^
	IPB^‡‡^	70.08 (67-75)	70.25 (68-76)	70.40 (68-75)	-0.16 (-1.97 to 1.64)	0.790^††^
Total Hb^§§^ (g/ dL^||||^)	IPA**	24.08 ±1.35	26.55 ±2.85	25.09 ±1.71	-1.00 (-3.00 to 0.98)	0.249^¶¶^
	IPB^‡‡^	25.12 ±1.98	26.09 ±1.99	27.09 ±2.29	-1.96 (-5.22 to 1.28)	0.181^¶¶^
Free Hb^§§^ (g/ dL^||||^)	IPA**	0.282 ±0.22	0.309 ±0.24	0.315 ±0.24	-0.03 (-0.05 to -0.07)	0.026^**¶¶**^
	IPB^‡‡^	0.298 ±0.20	0.312 ±0.21	0.313 ±0.20	0.02 (- 0.05 to 0.06)	0.922^¶¶^
Degree of hemolysis (%)	IPA**	0.36 ±0.27	0.35 ±0.29	0.38 ±0.30	-0.02 (-0.06 to 0.01)	0.222^¶¶^
	IPB^‡‡^	0.36 ±0.25	0.35 ±0.22	0.33 ±0.19	0.03 (-0.05 to 0.12)	0.345^¶¶^
Potassium (mmol/L***)	IPA**	39.0 ±2.1	38.5 ±2.2	39.9 ±2.7	-0.89 (-2.26 to 0.46)	0.151^¶¶^
	IPB^‡‡^	40.1 ±1.2	41.7 ±3,0	41.9 ±2.2	-1.87 (-3.41 to -0.33)	0.022^**¶¶**^

*IP- infusion pump; † C1- Control samples 1- collected from the packed red blood cell bag; ‡C2- Control samples 2-collected after the infusion lines were filled; §V-Velocity Sample- collected after infusion on IP in the selected rate;|| CI- Confidence Interval; ¶Ht- Hematocrit;**IPA- Infusion pump A; †† Friedman test (minimum -maximum); ‡‡IPB- Infusion Pump B; §§Hb- Hemoglobin; |||| g/dL- grams per deciliter;¶¶ Repeated measures ANOVA (Linear contrast between matters); ***mmol/L- millimol per liter.

There was no statistically significant difference in hematocrit, total hemoglobin concentration and degree of hemolysis, regardless of the IP used in the experiment. The free hemoglobin (*P*=0.026) and potassium (*P*=0.022) concentration presented significant increase when the RBC were submitted to IPA and IPB, respectively.Regarding the additional variables, the laboratory temperature ranged from 21.2ºC to 26.9ºC and the relative air humidity remained in the mean of 56.7 ± 2.9% during the experiments. The temperature of the blood components varied from 19.3ºC to 25.3ºC during the phases.

The storage time varied from 19 to 30 days, and the longest period was found in experiments with infusion rate of 300mL/h, both in IPA and in IPB. Regarding the exposition time of blood components to the environment in the experiment phases, the higher values were those at infusion rate of 100mL/h in IPB; the maximum value was 176 minutes.

## Discussion

The main findings of this study indicate that cellular injury occurred in some of the experiment phases due to the increase of hemoglobin and potassium markers.

Some studies have reported the possibility of cellular damage and extravascular hemolysis caused by transfusions held in mechanical infusion systems in view of increased hemoglobin in the plasma. Linear peristaltic infusion pumps are often found in hospitals and have advantages, such as flow control, time and volume monitoring. The use of IPs on RBC transfusion promotes greater patient safety because infusion time and volume can be accurately controlled, but some publications on hemolysis have reported conflicting results. Most of them describe the linear mechanism as the most prone to hemolytic events, suggesting that the damage caused to the erythrocytes may occur due to the device mechanism^7^ ^-^ ^8^.

The RBC quality control is a rigorous phase aimed at assessing the blood component preparation process. Quality is evaluated through previously set indicators by extrapolating the results of the referred production of the analyzed fraction. Quality control analyses in blood banks must be carried out in at least 1% of the production or in 10 units per month, according to the value representing most of the production. It is worth highlighting that not all blood component units transfused to patients are subjected to quality control assessment^9^ ^-^ ^10^ ^,^ ^15^.

The present study showed that the hematocrit and the total hemoglobin remained within the quality control standard. The degree of hemolysis, according to Brazilian legislation, is set at a maximum of 0.8%, for the last storage day of RBC preserved in CPDA-1 solution^19^. The reference values of potassium and free hemoglobin have not been stablished in literature, so they are not routinely assessed in blood banks. These markers were assessed in the present study according to their alteration during the experiment phases. Although free hemoglobin is not routinely assessed, the analysis thereof is an evaluation stage of degree of hemolysis in some analysis techniques used by blood banks^15^. 

The degree of hemolysis found in the experiments ranged from 0.06% to 0.83%. Two values above the recommended ones - 0.81% and 0.83% - were observed in the current study, both in the experiments using infusion rate at 300mL/h (Table 1). Data collection evidenced that the 0.83% value was found at the C1 moment. Despite the obtainment of blood products is well set by the sanitation and health authorities, there may be cases in which the blood component presents reference values different from those set by the quality control. The multidisciplinary team may not be aware of it, since the checking is not performed throughout the whole production. Thus, there is concern with the fact that the lack of analyses in markers throughout the whole production may result in damages to the patient.

The prolonged storage time of RBC could be an additional factor that contributes to increasing hemolysis markers. The intracellular concentration of potassium decreases as the storage time increases, whereas the extracellular concentration grows^16^ ^,^ ^20^ ^-^ ^21^. 

Studies have recommended that blood transfusions in some populations, such as newborns and children, must be performed with recently collected RBCs (at least seven days), in order to preserve the original features of the blood component and to avoid the damaging effects caused by its storage^23^ ^-^ ^24^. Potassium concentration presented statistically significant changes (*P* <0.010) (Table 2) in the present study and in the Bonferroni post-hoc test (Figure 1) due to the action of the programed infusion rate. When the markers were analyzed according to the selected flow (Table 3), potassium significantly increased at the rate of 300mL/h (p=0.021). Such flow can be associated with fast blood transfusions applied in severe hypovolemia cases and in hypercalcemia cases described in the literature. It is recommended RBC infusion of at most 5mL kg^-1^ minute^-1^ to avoid hypercalcemia^22^ ^-^ ^23^. Some publications suggest that hemolytic effects are more prone to occur in higher rates^8^ ^,^ ^17^.

The analyses of the markers according to the infusion pump (Table 4) presented statistically significant increase in the hemolysis markers, such as free hemoglobin in IPA (p=0.022) and potassium in IPB (p=0.026). These changes could be possibly explained by the infusion mechanism of the device. Other studies corroborate the potassium increase in experiments carried out with infusion pump and relate higher values to longer storage time^11^ ^-^ ^17^. A publication described extracellular potassium increase according to the storage time and to the infusion mechanism of the device, regardless of the selected flow^11^. High potassium levels used as hemolysis indicators have been described in studies associated with severe adverse events, such as arrhythmias and cardiocirculatory arrest, in massive and fast blood transfusion performed during emergency situations^21^ ^-^ ^23^.

The other markers, such as hematocrit, increased in all the phases of the conducted experiments, but such increase was not statistically significant. Hematocrit has been associated with the viscosity of the blood components in some publications; thus, the higher the hematocrit value is, the more viscous the blood product^18^ ^-^ ^19^. The free hemoglobin marker had increased in all experiment scenarios. These findings may lead to reflection about the occurrence of changes in the erythrocytes membrane from the mechanism of the infusion pump, infusion accessories and different infusion flows. Publication investigating non-immune-mediated hemoglobinuria in pediatric patients found greater results of free hemoglobin using a particular infusion pump, mainly in RBC units with higher hematocrit. Then, it was implemented a corrective action plan to prevent and minimize the risk of mechanical hemolysis by adopting RBC units with lower hematocrit and replacing their infusion pumps after analyzing the biomarkers^24^. 

Comparisons between the studied linear peristaltic IPs were not designed and this is a limitation of the current study. This option was made because of the small sample. There is, though, the need of extending the experiments in further studies to extrapolate the results. Additionally, other device-related variables are essential for the definition of the mechanism of action of devices on the RBCs, such as infusion and occlusion pressure; delimitation of the scenario applied to the device in order to assess the possible differences between manufacturers; and considerations about the environmental variables and blood components.

The variables related to the environment and to blood components are extremely important to the global assessment of markers in different moments of the experiment since they are possible confounding variables. They need to be analyzed in details. Thus, further studies about the theme must be conducted.

The development of further research is essential to substantiate the blood transfusion clinical practice and the nursing clinical practice of blood components administration. Detailed analyses of the variables related to IP, RBC and additional biomarkers, such as the lactate dehydrogenase, haptoglobin, among other injury markers, must be carried out.

There is, though, the need of extending the experiments in further studies to extrapolate the results. Additionally, the analysis of device-related variables are essential for the definition of the action of IPs over the RBC, such as infusion and occlusion pressures; delimitation of the scenario in order to assess the possible differences between manufacturers; besides considerations about the environmental and the blood components variables.

## Conclusion

Hemolysis risk induced by a linear peristaltic infusion pumps was identified in this study by an increase in free hemoglobin and potassium markers.

Potassium appeared to be an important parameter to assess the fragility of the plasmatic membrane of erythrocytes. There was also a significant increase in evolution of the experiments when it was assessed in all scenarios, as well as greater increase predisposition at flow of 300mL/h. As the potassium biomarker is often increased in aged packed red blood cells, we do not recommend using them in this scenario. Additional studies must be conducted on this theme.

## References

[1] Carson JL, Grossman BJ, Kleinman S, Tinmouth AT, Marques MB, Fung MK (2012). Red blood cell transfusion: a clinical practice guideline from
the AABB. Ann Intern Med.

[2] Müller MM, Geisen C, Zacharowski K, Ton T, Seifried E (2015). Transfusion of Packed Red Cells. Dtsch Arztebl Int.

[3] Poder TG Boileau JC, Lafrenière R Thibault L, Carrier N de Grandmont MJ (2017). Quantitative assessment of haemolysis secondary to modern
infusion pumps. Vox Sang.

[4] Heaton A (2009). Red blood cell hemolysis: an old standard in changing
times. Transfusion.

[5] Strobel E (2008). Hemolytic Transfusion Reactions. Transfus Med Hemother.

[6] Esfahani H, Dehghan A, Hosseini H, Esfahani S (201). In-Vitro Red Blood Cells Integrity and Morphology Changes after
Passing Through Volumetric Peristaltic Infusion Pump. IJBC.

[7] Nightingale MJ, Norfolk DR, Pinchon DJ (2010). Current uses of transfusion administration sets: a cause for
concern. Transfus Med.

[8] Gibson JS, Leff RD, Roberts RJ (1984). Effects of intravenous delivery systems on infused red blood
cells. Am J Hosp Pharm.

[9] Denison MUP, Bell R, Schuldreich R, Chaudhri MA (1991). Effect of different pump mechanisms on transfusion of
blood. Australas Phys Eng Sci Med.

[10] Frey B, Eber S, Weiss M (2003). Changes in red blood cell integrity related to infusion pumps: a
comparison of three different pump mechanisms. Pediatr Crit Care Med.

[11] Parfitt HS, Davies SV, Tighe P, Ewings P (2007). Red cell damage after pumping by two infusion control devices
(Arcomed VP 7000 and IVAC 572). Transfus Med.

[12] Lieshout-Krikke RW, Van der Meer PF, Koopman MMW, Korte DDE (2011). Effect on the quality of blood components after simulated blood
transfusions using volumetric infusion pumps. Transfusion.

[13] Gurdak RG, Anderson G, Mintz PD (1988). Evaluation of IVAC Variable Pressure Volumetric Pump Model 560
for the delivery of red blood cells, adenine-saline added. Am J Clin Pathol.

[14] Burch KJ, Phelps SJ, Constance TD (1991). Effect of an infusion device on the integrity of whole blood and
packed red blood cells. Am J Hosp Pharm.

[15] Hess JR, Sparrow RL, van der Meer PF, Acker JP, Cardigan RA, Devine DV (2009). Red blood cell hemolysis during blood bank storage: using
national quality management data to answer basic scientific
questions. Transfusion.

[16] Lacroix J, Tucci M (2011). Clinical impact of length of storage before red blood cell
transfusion. Transfus Clin Biol.

[17] Thompson HW, Lasky LC, Polesky HF (1986). Evaluation of a volumetric intravenous infusion pump for
transfusion of blood components containing red cells. Transfusion.

[18] Criss VR, DePalma L, Luban NL (1993). Analysis of a linear peristaltic infusion device for the
transfusion of red cells to pediatric patients. Transfusion.

[19] Carvalho EB, Borges EL, Carlos LMB, Silva MAM, Magalhães SMM, Gomes FVBAF (2007). Efeito da bomba de infusão de soluções sobre o grau de hemólise
em concentrado de hemácias. Rev Bras Hematol Hemoter.

[20] Holme S (2005). Current issues related to the quality of stored
RBCs. Transfus Apher Sci.

[21] Smith HM, Farrow SJ, Ackerman JD, Stubbs JR, Sprung J (2008). Cardiac Arrests Associated with Hyperkalemia During Red Blood
Cell Transfusion: A Case Series. Anesth Analg.

[22] Lee AC, Reduque LL, Luban NLC, Ness PM, Anton B, Heitmiller ES (2014). Transfusion-associated hyperkalemic cardiac arrest in pediatric
patients receiving massive transfusion. Transfusion.

[23] Strauss RG (2010). Red blood cell storage and avoiding hyperkalemia from
transfusions to neonates and infants. Transfusion.

[24] Hughes J, McNaughton J, Andrews J, George T, Bergero C, Pyke-Grimm K (2015). Infusion Pump-Mediated Mechanical Hemolysis in Pediatric
Patients. Ann Clin Lab Sci.

